# Prognostic Impact of C-reactive Protein-Albumin-Lymphocyte Index in Patients Who Underwent Splenectomy and Devascularization for Gastric Varices Caused by Portal Hypertension

**DOI:** 10.7759/cureus.87092

**Published:** 2025-07-01

**Authors:** Ko Oshita, Tsuyoshi Kobayashi, Naruhiko Honmyo, Seiichi Shimizu, Shintaro Kuroda, Hiroyuki Tahara, Masahiro Ohira, Kentaro Ide, Hideki Ohdan

**Affiliations:** 1 Department of Gastroenterological and Transplant Surgery, Hiroshima University Hospital, Hiroshima, JPN

**Keywords:** c-reactive protein-albumin-lymphocyte index, gastric varices, portal hypertension, splenectomy, systemic inflammation

## Abstract

Purpose: The C-reactive protein-albumin-lymphocyte (CALLY) index is a novel nutritional and inflammation-based index. This study aimed to evaluate the prognostic value of the preoperative CALLY index in patients who underwent splenectomy and devascularization (Sp + Dev) for gastric varices caused by portal hypertension.

Methods: This study included 64 patients who underwent Sp + Dev for gastric varices between January 2009 and March 2022. The CALLY index was calculated as (albumin × lymphocyte)/(C-reactive protein × 10^4^), and the patients were divided into high and low CALLY groups. Log-rank tests were performed to compare the overall survival (OS). Independent risk factors for OS were identified by multivariate analysis. Propensity score-matching was performed for survival analysis to balance the selection bias for stratification of the CALLY index.

Results: The cut-off value of the CALLY index was set at 4.35 using receiver-operating characteristic curve analysis, and 39 patients were classified into the low CALLY group. The low CALLY group had significantly worse OS (p = 0.013) than the high CALLY group. Multivariate analysis identified a low CALLY index (hazard ratio (HR) = 3.787; 95% confidence interval (CI) = 1.174-12.218; p = 0.026) and concurrent hepatocellular carcinoma (HR = 2.914; 95% CI = 1.018-8.342; p = 0.046) as independent risk factors for poor OS. After propensity score-matching to balance the selection bias, a low CALLY index significantly correlated with worse OS (p = 0.026). The low CALLY group had significantly worse portal vein pressure after splenectomy than the high CALLY group (p = 0.031).

Conclusions: Preoperative CALLY index is a significant prognostic indicator for gastric varices after Sp + Dev. The CALLY index may be useful in determining the suitability of surgical treatment and perioperative management.

## Introduction

Gastric varices (GVs) are complications of portal hypertension and are present in approximately 20% of patients with liver cirrhosis [1]. Gastric variceal bleeding is a life-threatening condition associated with extremely high mortality rates (up to 45%) owing to its large shunt diameter and high flow volume [1,2]. Interventional radiology, including balloon-occluded retrograde transvenous obliteration (BRTO) and transjugular intrahepatic portosystemic shunt, is generally performed for GVs management. Splenectomy and devascularization (Sp + Dev) are also established treatment procedures that can improve GVs and portal hypertension and are chosen for patients who have difficulty with interventional radiology, hypersplenism, or severe portal hypertension [3,4]. Since Sp + Dev is relatively invasive due to poor liver function and hypersplenism, identifying preoperative prognostic factors is essential for surgeons. However, there are few reports on prognostic factors for GVs, and it is important to identify preoperative prognostic biomarkers for patients who underwent Sp + Dev for GVs.

Nutritional and inflammatory statuses are key factors that are strongly associated with the prognosis of liver cirrhosis. In recent years, several nutritional and inflammation-based indices calculated from blood examinations have been proposed and demonstrated to be associated with the prognosis of liver cirrhosis [5-8]. The C-reactive protein (CRP)-albumin-lymphocyte (CALLY) index, which combines nutrition, inflammation, and immune system status, has been reported as a novel prognostic scoring system [9]. This index was initially developed as a prognostic predictor for patients who underwent hepatectomy for hepatocellular carcinoma (HCC) and has since then been demonstrated to have superior prognostic ability than other indices in various cancers [10-12].

Each of the three factors comprising the CALLY index is known to be related to the prognosis of liver cirrhosis and the severity of portal hypertension. Previously reported indices consisted of two factors, whereas the CALLY index, which combines three factors associated with liver cirrhosis and portal hypertension, may provide a more comprehensive representation of nutrition and inflammatory status. Thus, we hypothesized that the preoperative CALLY index could predict the outcome of patients who underwent Sp + Dev for GVs caused by portal hypertension. The correlation between the CALLY index and the prognosis of portal hypertension or liver cirrhosis has not been explored. This study aimed to evaluate the effect of preoperative CALLY index on the prognosis of GVs after Sp + Dev.

## Materials and methods

This retrospective study was performed in accordance with the Declaration of Helsinki and approved by the Institutional Review Board of Hiroshima University (E-1746). Informed consent was obtained from all the patients.

Patients

Clinical data of patients who underwent Sp + Dev for GVs at Hiroshima University Hospital between January 2009 and March 2022 were retrospectively collected. GVs were diagnosed using upper gastrointestinal endoscopy in all cases. The indication for treatment was the occurrence of red signs, F3, or rapidly enlarging varicose veins with a high risk of rupture. Difficulty with interventional radiology and endoscopic therapy, thrombocytopenia due to hypersplenism, or severe portal hypertension were indications for Sp + Dev. Surgery was not indicated for portal vein thrombosis, refractory ascites, and Child-Pugh grade C. Uncontrolled concurrent HCC was also excluded as a surgical indication, and HCC was evaluated using the tumor-nodes-metastasis (TNM) 8th edition classification.

Surgical procedure and postoperative follow-up

Spleen mobilization was performed using hand-assisted laparoscopic surgery, and splenic vessel dissection and devascularization were performed via laparotomy using a midline incision. The patient was placed in the supine position, and an upper midline incision of 8.0 cm was made. A 12-mm trocar was inserted into the caudal side of the umbilicus for the laparoscope, and a 5-mm trocar was inserted into the left side of the abdomen. After a hand port (Gelport; Applied Medical, Rancho Santa Margarita, CA, USA) was introduced into the midline incision, the abdomen was insufflated with carbon dioxide to 10 cmH_2_O. The splenocolic ligament, gastrosplenic ligament, and retroperitoneal attachments were dissected using a Thunderbeat (Olympus Co., Tokyo, Japan), and the spleen was mobilized. Subsequently, a median upper incision was extended to the incision on the caudal side of the umbilicus, and subsequent procedures were performed via open laparotomy. The gastrocolic ligament was dissected, and the omental bursa was opened. The splenic artery and vein were ligated, and the spleen was removed. After splenectomy, the vessels of the greater and lesser curvatures were devascularized. The portal vein pressure was measured using a transducer inserted through the jejunal vein during surgery to determine the extent of devascularization. The surgical procedure followed for open Sp + Dev was as previously described [13].

Perioperative clinical data, including operative time, blood loss, postoperative complications, mortality, and length of postoperative hospital stay, were collected. Postoperative complications were categorized according to the Clavien-Dindo (CD) classification. As a follow-up for GVs, upper gastrointestinal endoscopy was performed within 3 months after discharge and every 6 months or 1 year after surgery, or when indicated clinically.

Definitions

Blood samples were collected within 14 days before surgery. The prognostic nutritional index (PNI), CALLY index, neutrophil count to albumin ratio (NAR), neutrophil to lymphocyte ratio (NLR), lymphocyte to monocyte ratio (LMR), and platelet to lymphocyte ratio (PLR) were calculated as follows:

PNI = serum albumin level (g/dL) × 10 + lymphocyte count (/mm^3^) × 0.005.

CALLY index = (serum albumin level (g/dL) × lymphocyte count)/(serum CRP level (mg/dL) × 10^4^).

NAR = neutrophil count (10^9^/L)/serum albumin level (mg/dL).

NLR = neutrophil count/lymphocyte count.

LMR = lymphocyte count/monocyte count.

PLR = platelet count/lymphocyte count.

Albumin-bilirubin score was classified according to the modified albumin-bilirubin (mALBI) grading system [14].

Statistical analysis

Continuous variables are expressed as mean ± standard deviation, and categorical variables as numbers and percentages. To compare the two groups, the student’s t-test was performed for continuous variables, and the Chi-square test or Fisher’s exact test were performed for categorical variables. The cut-off values for each index were set using receiver-operating characteristic (ROC) curve analysis of survival status at the 5-year follow-up. Survival analysis was performed using the Kaplan-Meier method and compared between different groups using the log-rank test. Overall survival (OS) was defined as the time between the date of Sp + Dev and the date of death from any cause. Univariate and multivariate analyses were performed using the Cox proportional hazards model, and the relevant univariate variables (p < 0.100) were included in the multivariate analysis. Propensity score-matching (PSM) analysis was performed to overcome the bias caused by the different distributions of covariates between the high and low CALLY groups. Propensity score-matched analysis was performed based on baseline characteristics such as age, sex, etiology of hepatitis, presence of concurrent HCC, and major organ function indicators such as serum creatinine level, mALBI grade, and platelet count. One-to-one matching was performed using a 0.25 caliper. Dynamics in portal vein pressure before and after splenectomy and after Sp +Dev, and changes in liver function and platelet counts before and after Sp + Dev were assessed using a paired t-test. All statistical analyses were performed using JMP software (version 18.0; SAS Institute Inc., Cary, NC, USA), and statistical significance was set at p < 0.05.

## Results

Baseline characteristics

During the study period of January 2009-March 2022, 64 patients underwent Sp + Dev for GVs. No patients were excluded; all 64 patients who underwent Sp + Dev were included in this study. The demographic characteristics are presented in Table 1. The mean age was 62.6 ± 11.5 years; the numbers of male and female patients were 46 (71.9%) and 18 (28.1%). Sixty-three patients received prophylactic treatment for GVs (98.4%), and 27 had concurrent HCC (42.0%). Postoperative complications ≥ CD grade 3 occurred in nine patients (14.1%).

**Table 1:** Baseline characteristics of patients. Variables are expressed as mean ± SD or number (%) ALBI: albumin-bilirubin; CALLY: C-reactive protein-albumin-lymphocyte; CD: Clavien-Dindo; LMR: lymphocyte to monocyte ratio; MELD: model for end-stage liver disease; mGPS: modified Glasgow prognostic scale; NAR: neutrophil to albumin ratio; NLR: neutrophil to lymphocyte ratio; PLR: platelet to lymphocyte ratio; PNI: prognostic nutrition index; TNM: tumor-nodes-metastasis.

Variables	n = 64
Age (years)	62.6 ± 11.5
Sex	
Male	46 (71.9%)
Female	18 (28.1%)
Viral hepatitis	35 (54.7%)
Diabetes mellitus	15 (23.4%)
Prophylactic treatment	63 (98.4%)
Concurrent hepatocellular carcinoma	27 (42.0%)
TNM stage II–IV	57 (89.1%)
White blood cell counts (10^9^/L)	3.46 ± 1.79
Neutrophil counts (10^9^/L)	2.21 ± 1.53
Lymphocyte counts (10^9^/L)	0.87 ± 0.38
Monocyte counts (10^9^/L)	0.24 ± 0.11
Total bilirubin (mg/dL)	1.3 ± 0.6
Albumin (g/dL)	3.6 ± 0.5
Prothrombin time activity (%)	69.5 ± 14.1
Platelet counts (10^9^/L)	70.2 ± 41.7
Aspartate aminotransferase (IU/L)	38.3 ± 22.2
Alanine aminotransferase (IU/L)	29.9 ± 2.5
Cholinesterase (IU/L)	163.9 ± 52.3
Creatinine (mg/dL)	0.89 ± 1.23
C-reactive protein (mg/dL)	0.19 ± 0.30
Child-Pugh score	6.6 ± 1.2
MELD score	10.3 ± 3.2
ALBI score	-2.15 ± 0.46
mGPS score	
0	33 (51.6%)
1	28 (12.5%)
2	3 (4.7%)
PNI	39.9 ± 5.4
CALLY index	5.22 ± 4.90
NAR	0.6 ± 0.4
NLR	2.8 ± 1.8
LMR	4.0 ± 1.8
PLR	85.8 ± 42.1
Operative time (min)	292.6 ± 86.5
Blood loss (mL)	934.3 ± 1208.7
Postoperative complications CD grade 1–2	49 (76.6%)
Ascites	28 (44.4%)
Portal vein thrombosis	13 (20.3%)
Delayed gastric emptying	5 (7.8%)
Others	3 (4.7%)
Postoperative complications CD ≥ grade 3	9 (14.1%)
Intraabdominal abscess	5 (7.8%)
Intraabdominal bleeding	4 (6.3%)
Pancreatic fistula	2 (3.1%)
Bleeding of esophageal varices	1 (1.6%)
Perforation of stomach	1 (1.6%)
Re-operation	4 (6.3%)
90-day mortality	1 (1.6%)
Postoperative hospital stay (days)	18.0 ± 2.2

**Figure 1:**
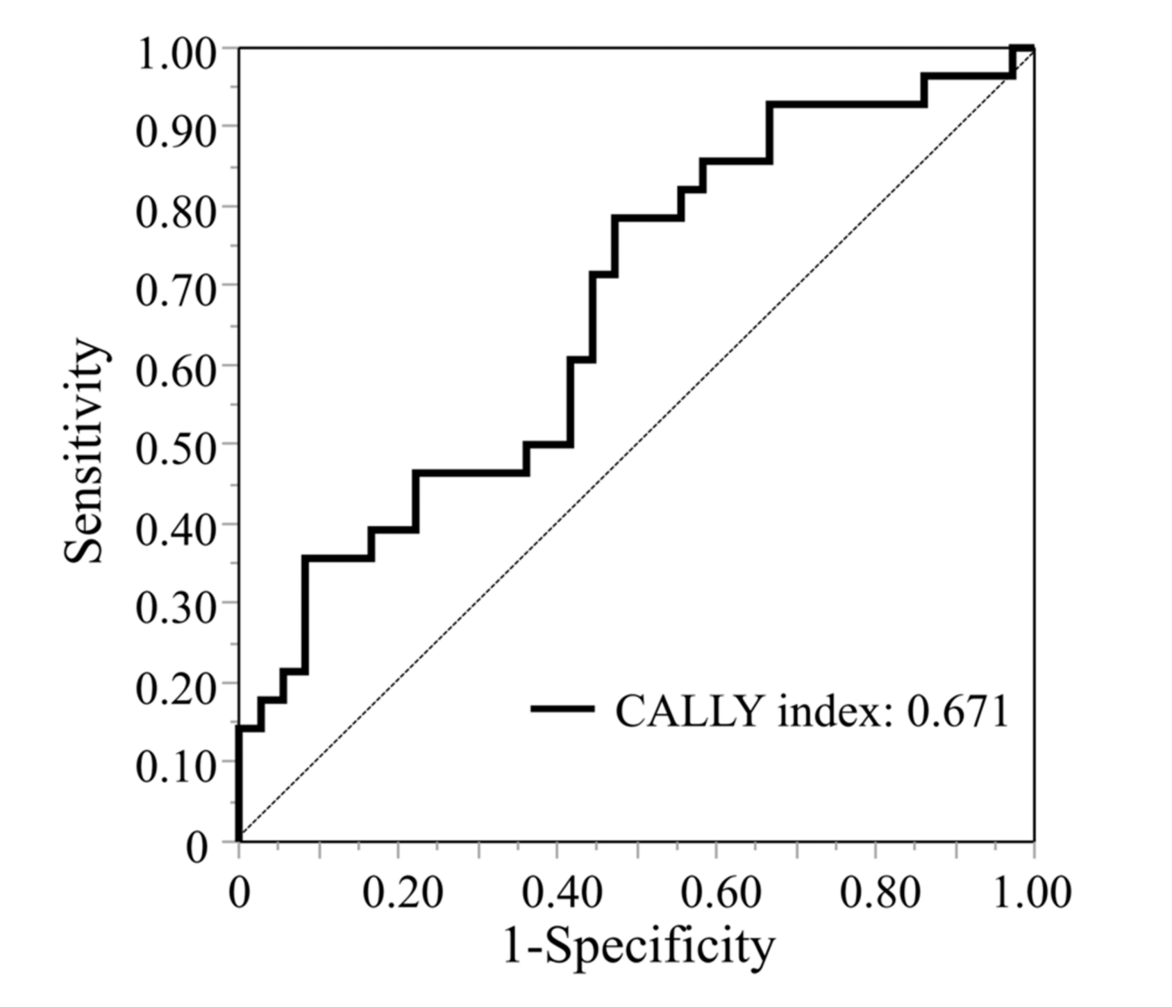
Area under the ROC curve for outcome prediction for the CALLY index The area under the ROC curve of the CALLY index was 0.671. CALLY: C-reactive protein-albumin-lymphocyte; ROC: receiver-operating characteristic

The optimal cut-off values for each index were set using the area under the curve (AUC): PNI: 36.4, AUC: 0.534; CALLY index: 4.35, AUC; 0.671; NAR: 0.7, AUC: 0.599; NLR: 1.9, AUC: 0.594; LMR: 2.9, AUC: 0.561; PLR: 62.6, AUC: 0.609 and model for end-stage liver disease (MELD) score: 7.9, AUC: 0.456. Figure 1 shows the ROC curve of the CALLY index at 5 years after surgery. Of the 64 patients, 39 (60.9%) had a CALLY index of less than 4.35 and were categorized into the low CALLY group.

Survival analysis and risk factors for poor prognosis

**Figure 2:**
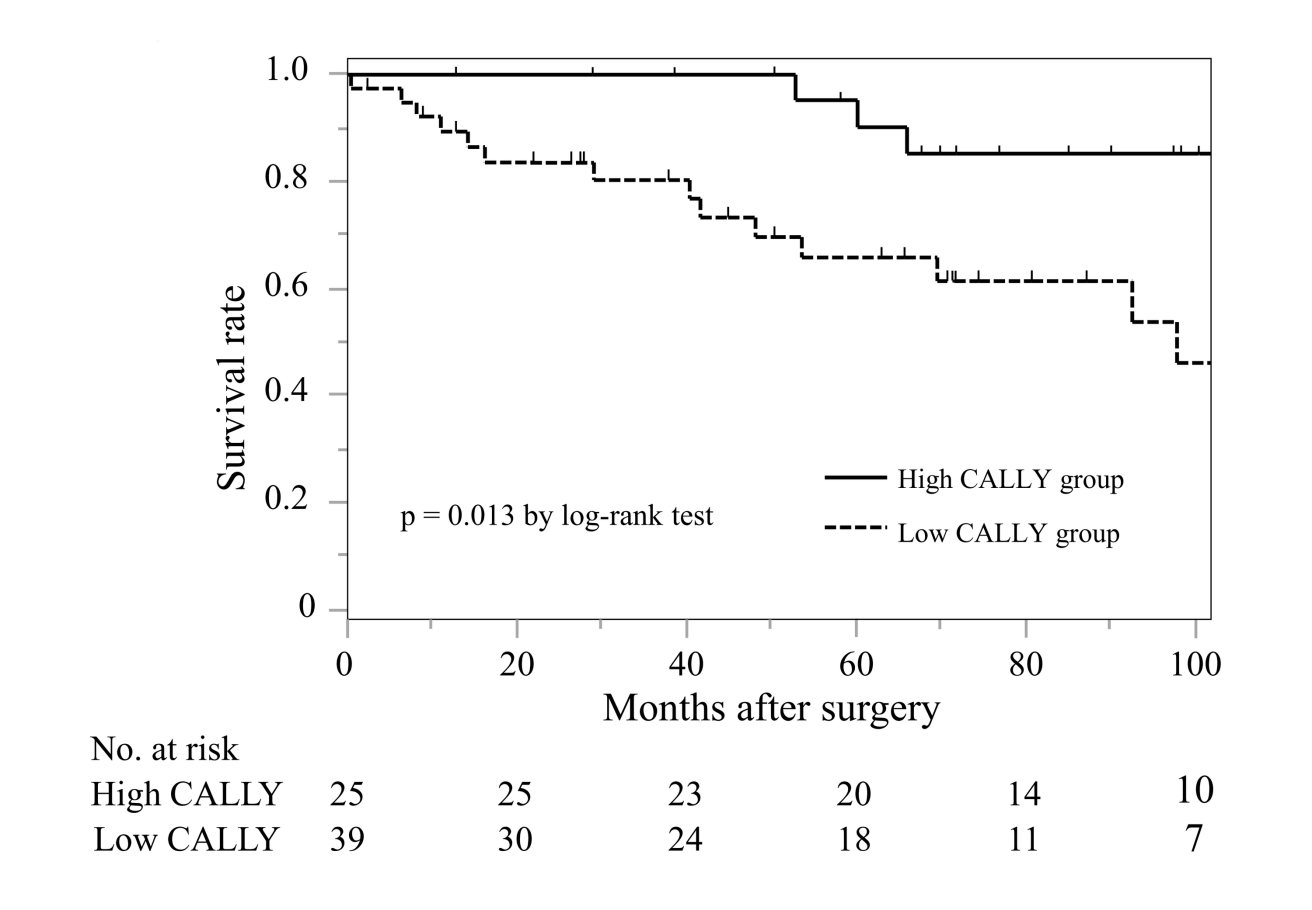
Survival analysis classified according to the CALLY index in patients who underwent Sp + Dev for gastric varices. Overall survival was significantly worse in the low CALLY group than the high CALLY group (p = 0.013). CALLY: C-reactive protein-albumin-lymphocyte; Sp + Dev: splenectomy + devascularization

The mean follow-up period was 66.9 ± 41.3 months, and the 5-year OS rate was 76.0%. As shown in Figure 2, the low CALLY group had significantly poorer OS (5-year OS, 90.2% vs. 69.6%, p = 0.013) compared with the high CALLY group. Eight patients (12.5%) developed GVs requiring re-treatment; BRTO was performed in five patients, and endoscopic injection sclerotherapy in three patients.

Table 2 shows the results of univariate and multivariate analyses of prognostic factors for OS. In the univariate analysis, diabetes mellitus, concurrent HCC, mALBI ≥ grade 2b, low PNI, and a low CALLY index were associated with poor OS. In the multivariate analysis, low CALLY index (hazard ratio (HR) = 3.787, 95% confidence interval (CI) 1.174-12.218; p = 0.026) and concurrent HCC (HR = 2.914, 95% CI 1.018-8.342; p = 0.046) were identified as independent predictors for OS.

**Table 2:** Prognostic factors for overall survival identified by univariate and multivariate analyses. CALLY: C-reactive protein-albumin-lymphocyte; CI: confidential interval; HR: hazard ratio; LMR: lymphocyte to monocyte ratio; mALBI: modified albumin-bilirubin; mGPS: modified Glasgow prognostic scale; NAR: neutrophil to albumin ratio; NLR: neutrophil to lymphocyte ratio; PLR: platelet to lymphocyte ratio; PNI: prognostic nutrition index

Variables		Univariate Analysis			Multivariate Analysis		
	n (%)	HR	95% CI	p-value	HR	95% CI	p-value
Age ≥ 70 (years)	18 (28.1%)	2.108	0.815-5.451	0.124			
Sex, male	46 (71.9%)	1.572	0.521–4.750	0.418			
Viral hepatitis	29 (45.3%)	1.438	0.581–3.559	0.430			
Diabetes mellitus	15 (23.4%)	2.663	1.050–6.759	0.032	2.164	0.827–5.666	0.116
Concurrent hepatocellular carcinoma	27 (42.2%)	3.635	1.364–9.690	0.006	2.914	1.018–8.342	0.046
Creatinine ≥ 0.80 (mg/dL)	21 (32.8%)	1.16	0.438–3.073	0.765			
Child-Pugh class B	35 (54.8%)	1.231	0.496–3.053	0.654			
MELD score ≥ 7.9	56 (87.5%)	1.464	0.334–6.411	0.611			
mALBI ≥ Grade 2b	39 (60.9%)	2.703	0.893–8.184	0.078	1.043	0.281–3.872	0.949
mGPS 1-2	31 (48.4%)	1.219	0.491–3.024	0.669			
PNI ≤ 36.4	48 (75.0%)	2.479	0.878–6.996	0.076	2.213	0.701–6.988	0.176
CALLY index ≤ 4.35	25 (39.1%)	3.701	1.223–11.203	0.013	3.787	1.174–12.218	0.026
NAR ≥ 0.7	15 (23.4%)	1.801	0.638–5.085	0.260			
NLR ≤ 1.9	43 (67.2%)	1.022	0.400–2.609	0.964			
LMR ≥ 2.9	44 (68.8%)	1.425	0.529–3.841	0.482			
PLR ≤ 62.6	44 (68.8%)	1.813	0.650–5.061	0.249			
Operative time ≥ 300 (min)	26 (40.6%)	1.712	0.775–3.783	0.300			
Blood loss ≥ 1000 (mL)	18 (28.1%)	1.578	0.693–3.593	0.273			

In the subgroup analysis, the low CALLY group tended to show poorer OS in both the concurrent HCC group (n = 37, p = 0.069) and the non-concurrent HCC group (n = 27, p = 0.083).

Comparison between high and low C-reactive protein-albumin-lymphocyte (CALLY) groups and survival analysis using propensity score-matching

**Figure 3:**
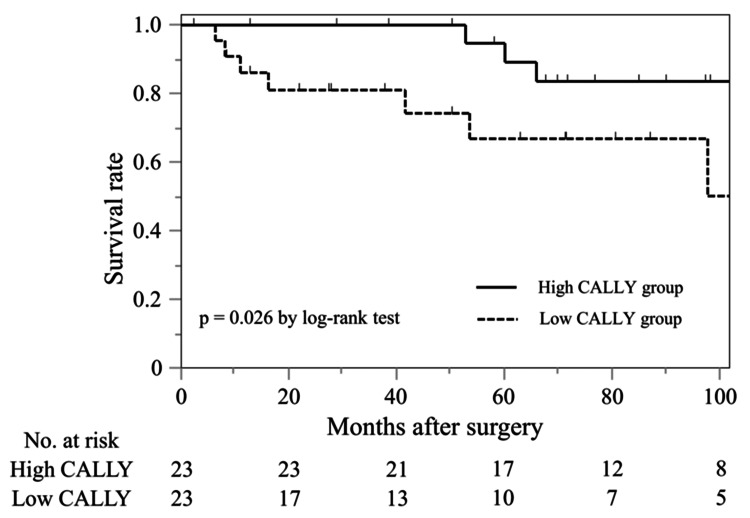
Survival analysis classified according to the CALLY index after propensity score-matching in patients who underwent Sp + Dev for gastric varices. Overall survival was significantly worse in the low CALLY group than the high CALLY group (p = 0.026) after propensity score-matching. CALLY: C-reactive protein-albumin-lymphocyte; Sp + Dev: splenectomy + devascularization

Baseline characteristics were compared between the high and low CALLY groups in Table 3. Before PSM, the proportions of male patients (p = 0.093), those with viral hepatitis (p = 0.084), and those with mALBI ≥ grade 2b (p = 0.090) tended to be higher in the low CALLY group. There were no significant differences in the proportions of patients with concurrent HCC (p = 0.776) or HCC ≥ TNM stage II (p = 0.653) between the two groups. After PSM, 23 patients were matched in each group, and the distribution of all clinical characteristics was balanced between the two groups. As shown in Figure 3, the low CALLY group showed a significantly worse OS (p = 0.026) even after PSM.

**Table 3:** Comparison of the clinical variables between the high and low CALLY groups before and after propensity score-matching. Variables are expressed as mean ± SD or number (%) CALLY: C-reactive protein-albumin-lymphocyte; MELD: model for end-stage liver disease; mALBI: modified albumin-bilirubin; MELD: model for end-stage liver disease; TNM: tumor-nodes-metastasis

Variables	Whole Study Series			Propensity Score-Matched Series		
	High CALLY group (n = 25)	Low CALLY group (n = 39)	p-value	High CALLY group (n = 23)	Low CALLY group (n = 23)	p-value
Age (years)	62.3 ± 12.4	62.9 ± 11.0	0.842	60.6 ± 12.9	61.6 ± 13.1	0.854
Sex, Male	15 (60.0%)	31 (79.5%)	0.093	15 (65.2%)	17 (73.9%)	0.521
Viral hepatitis	17 (68.0%)	18 (46.2%)	0.084	16 (69.6%)	11 (47.8%)	0.134
Diabetes mellitus	5 (20.0%)	10 (25.6%)	0.601	5 (21.7%)	6 (26.1%)	0.730
Concurrent hepatocellular carcinoma	10 (40.0%)	17 (43.5%)	0.776	10 (43.5%)	7 (30.4%)	0.360
TNM stage ≥ II	7 (36.8%)	13 (33.3%)	0.653	7 (30.4%)	6 (26.1%)	0.522
Platelet counts (10^9^/L)	73.6 ± 48.4	68.1 ± 37.3	0.614	77.3 ± 53.7	64.3 ± 33.7	0.695
Aspartate aminotransferase (IU/L)	41.1 ± 30.0	36.4 ± 15.5	0.412	35.2 ± 20.6	32.5 ± 11.2	0.289
Alanine aminotransferase (IU/L)	33.0 ± 27.9	27.9 ± 12.0	0.319	26.5 ± 19.2	26.2 ± 11.3	0.132
Cholinesterase (IU/L)	168.5 ± 54.7	161.0 ± 51.3	0.580	169.1 ± 57.8	160.5 ± 56.7	0.805
Creatinine (mg/dL)	1.1 ± 0.4	0.8 ± 0.2	0.301	1.2 ± 0.5	0.7 ± 0.1	0.393
Child-Pugh class B	14 (56.0%)	21 (53.8%)	0.856	13 (56.5%)	11 (47.8%)	0.555
MELD score	10.4 ± 4.7	10.3 ± 2.1	0.865	10.7 ± 5.0	9.9 ± 1.6	0.956
mALBI ≥ grade 2b	12 (48.0%)	27 (69.3%)	0.090	12 (52.1%)	13 (56.5%)	0.767

Surgical and short-term outcomes between high and low CALLY groups

Surgical and short-term outcomes were compared between the high and low CALLY groups in Table 4. Although intraoperative blood loss was significantly higher in the low CALLY group (p = 0.043), there was no significant difference in postoperative complications and hospital stay between the two groups.

**Table 4:** Comparison of surgical and short-term outcomes between the high and low CALLY groups Variables are expressed as mean ± SD or number (%) CALLY: C-reactive protein-albumin-lymphocyte; CD: Clavien-Dindo

Variables	High CALLY Group (n = 25)	Low CALLY Group (n = 39)	p-value
Operative time (min)	270.5 ± 57.7	307.2 ± 99.1	0.100
Blood loss (mL)	553.8 ± 78.8	1178.1 ± 235.8	0.043
Any operative complications	22 (88.0%)	34 (87.2%)	0.923
Postoperative complications CD ≥ grade 2	22 (88.0%)	29 (74.4%)	0.173
Postoperative complications CD ≥ grade 3	3 (12.0%)	6 (15.4%)	0.702
Re-operation	2 (8.0%)	2 (5.1%)	0.643
90-day mortality	0 (0.0%)	1 (2.6%)	0.420
Postoperative hospital stay (days)	17.6 ± 3.8	18.3 ± 2.6	0.882

**Figure 4:**
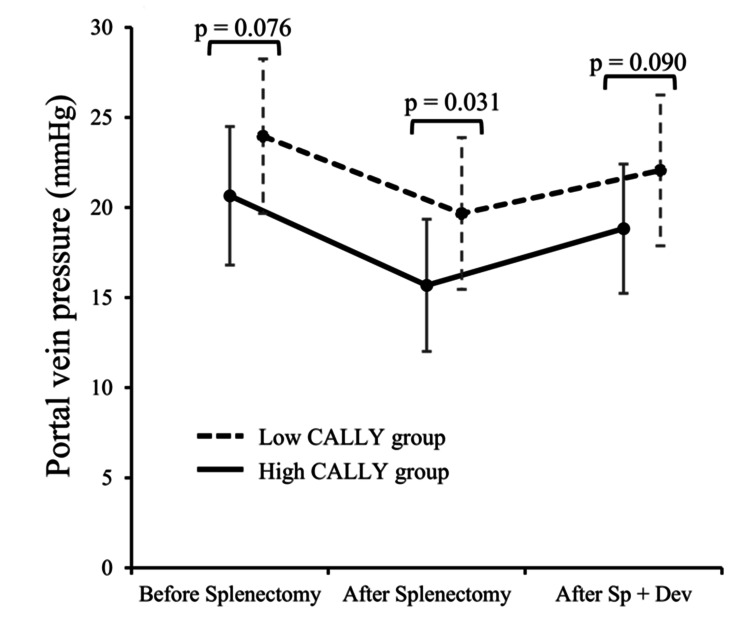
Changes in portal vein pressure before and after splenectomy and after Sp + Dev in the low and high CALLY index groups. Changes in portal vein pressure before and after splenectomy and after Sp + Dev in the low and high CALLY index groups are shown. Variables are expressed as the mean ± SD. CALLY: C-reactive protein-albumin-lymphocyte; Sp + Dev: splenectomy and gastric devascularization

Figure 4 shows the portal vein pressure before and after splenectomy in the high and low CALLY groups. In the low CALLY group, portal vein pressure tended to be higher than in the high CALLY group both before splenectomy (20.6 ± 3.8 mmHg vs 22.7 mmHg ± 4.5 mmHg, p = 0.076) and after Sp + Dev (18.8 ± 3.6 mmHg vs 20.7 ± 4.4 mmHg, p = 0.090). The low CALLY group had significantly higher portal vein pressure than the high CALLY group after splenectomy (15.7 ± 3.7 mmHg vs 18.2 ± 4.4 mmHg, p = 0.031). In both groups, a significant decrease in portal vein pressure after Sp + Dev was observed using a paired t-test (p < 0.001).

Changes in liver function

**Figure 5:**
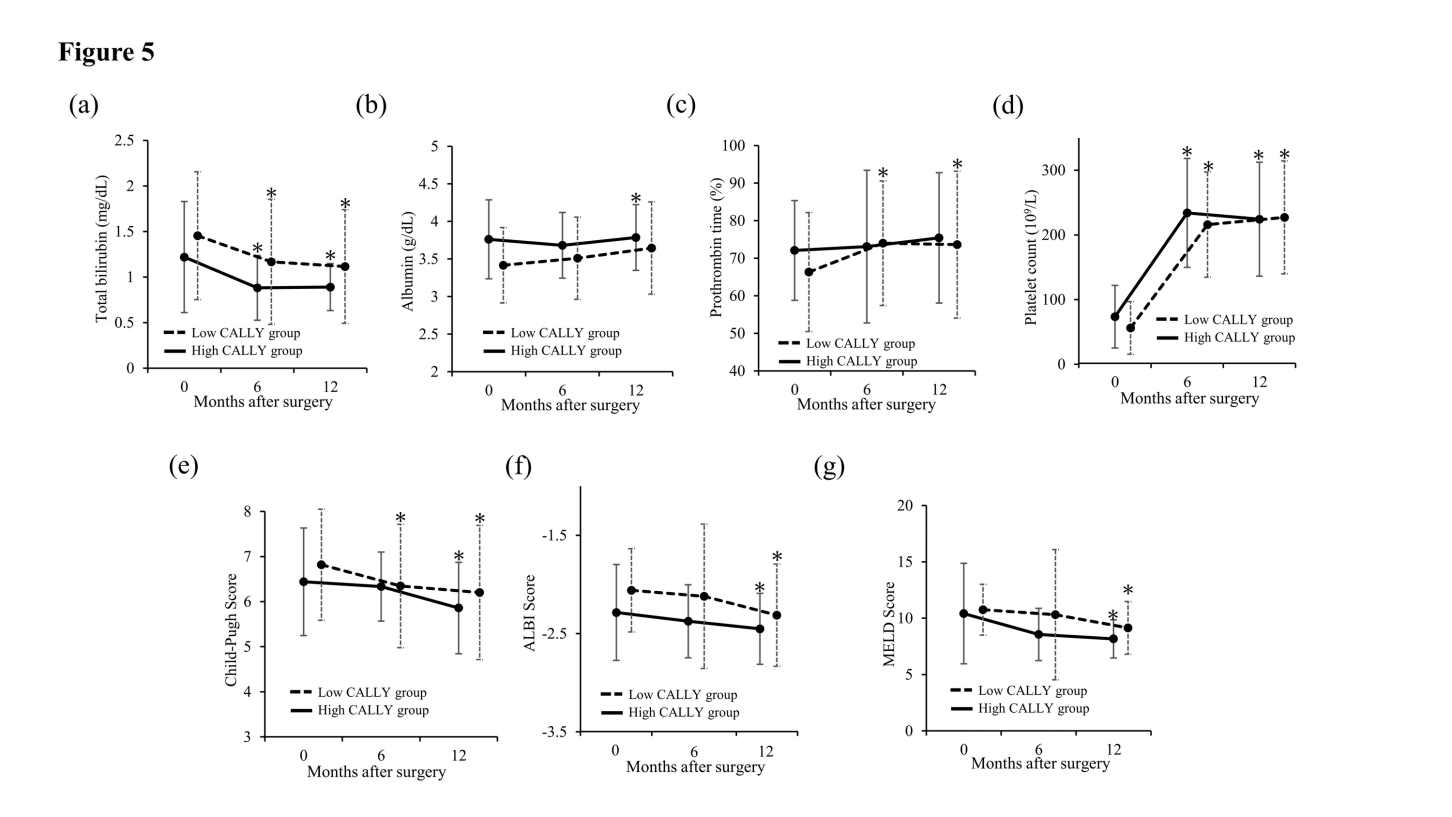
Changes in liver function after Sp + Dev in the low and high CALLY groups Changes in liver function after Sp + Dev in the low and high CALLY groups assessed by paired t-test: changes in a) total bilirubin, b) albumin, c) prothrombin time%, d) platelet counts, e) Child-Pugh score, f) ALBI score, g) MELD score differed by procedure over time. Variables are expressed as the mean ± SD. ALBI: albumin-bilirubin; CALLY: C-protein-albumin-lymphocyte; MELD: model for end-stage liver disease; Sp + Dev: splenectomy and gastric devascularization *p <0.050

Changes in liver function before and after Sp + Dev at 6 and 12 months are shown in Figure 5. Six months after Sp + Dev, total bilirubin level (p <0.050) and platelet counts (p < 0.001) were significantly improved in both groups, and the low CALLY group showed a significant decrease in Child-Pugh score (p = 0.025). Twelve months after Sp + Dev, the high CALLY group demonstrated significant improvements in total bilirubin level (p = 0.011), albumin level (p = 0.025), platelet count (p <0.001), Child-Pugh score (p = 0.048), ALBI score (p = 0.004) and MELD score (p = 0.041) were significantly improved in the high CALLY groups. In the low CALLY group, total bilirubin level (p = 0.001), prothrombin time (p = 0.021), platelet count (p < 0.001), Child-Pugh score (p = 0.033), ALBI score (p = 0.025) and MELD score (p <0.001) significantly improved at 12 months after Sp + Dev.

## Discussion

Nutritional and inflammation-based indices are novel prognostic biomarkers that combine systemic inflammation, nutritional or immune status, and have been applied to various diseases in recent years. However, there were no previous reports on the relationship between nutritional and inflammation-based indices and prognosis after Sp + Dev in GVs. In this study, preoperative CALLY index demonstrated significant prognostic value for OS in patients who underwent Sp + Dev for GVs caused by portal hypertension.

The CALLY index is a prognostic biomarker consisting of serum CRP level as an indicator of systemic inflammation (SI), serum albumin level as an indicator of nutritional status, and lymphocyte count as an indicator of immune status. SI has been shown to cause serious complications associated with portal hypertension and is associated with poor outcomes in liver cirrhosis with or without bacterial infections [15], CRP is mainly synthesized during the acute phase of inflammation in response to interleukin 6, which is associated with the activation of the inflammatory response in liver cirrhosis [16]. Elevated CRP levels can diagnose SI and are significantly correlated with poor prognosis in liver cirrhosis [17]. Albumin is a well-recognized indicator of nutrition and liver function and one of the most frequent significant prognostic predictors of liver cirrhosis [18]. Lower albumin level has been reported as an independent poor prognostic factor after treatment for GVs [4]. Lymphocytes have been identified as factors that reflect the immune response state in chronic liver disease. In liver cirrhosis, the absolute number of lymphocytes decreases, and immune dysfunction occurs due to the dysfunction of memory B cells and depletion of helper and cytotoxic T cells [19]. A decreased lymphocyte count is a predictor of prognosis in patients listed for liver transplantation [20]. Each of the three components of the CALLY index is closely related to the prognosis of liver cirrhosis, suggesting that the CALLY index may have a favorable ability to estimate the prognosis of GVs.

Low CALLY index and concurrent HCC were significant risk factors for OS in this study. HCC often occurs in patients with portal hypertension, and several reports have also described concurrent HCC as an independent prognostic factor after treatment for GVs [21, 22]. Improvement in thrombocytopenia due to hypersplenism and liver function following Sp + Dev has a significant impact on subsequent treatment for HCC, and concurrent HCC in GVs treatment should be considered when evaluating prognosis. Although the Child-Pugh classification, ALBI grade, and MELD score are reliable modalities for stratifying liver cirrhosis based on mortality risk, these scores were not identified as significant poor prognostic factors in this study. The present study included patients with relatively better liver function, excluding those with Child-Pugh class C or refractory ascites, as candidates for Sp + Dev. In addition, this study showed significant improvement of liver function, including Child-Pugh score, ALBI score and MELD score, were observed after Sp + Dev in both low and high CALLY index groups. Several surgeons have previously demonstrated the effect of splenectomy on improving liver function in liver cirrhosis, and splenectomy was useful for the conduct of interferon therapy for hepatitis C virus (HCV) patients with hypersplenism [23, 24]. Improvement in liver function and hypersplenism after Sp + Dev may have made it difficult to predict the prognosis based on preoperative liver function. In addition, surgical outcomes, including longer operative time and increased blood loss, were not associated with poor OS in this study.

Although the mechanism by which the CALLY index is related to prognosis remains unclear, a low CALLY index has been described as possibly associated with poor nutrition and cancer progression in cancer treatment [10, 12]. In patients with HCC, a low CALLY index reflects poor liver function and cancer progression [9]. C-reactive protein (CRP)-to-albumin ratio (CAR), NAR, and NLR have been reported to be associated with poor liver function in patients with liver cirrhosis, and nutritional and inflammation-based indices may play a role in liver function in liver disease [6-8]. In this study, a low CALLY index was significantly associated with high portal vein pressure after splenectomy and tended to be associated with poor portal vein pressure before splenectomy and after Sp + Dev, as well as mALBI grade. SI induces a disequilibrium between vasoconstrictor and vasodilator mechanisms in the liver and increases portal vein pressure [25]. Several studies have demonstrated that an elevated hepatic venous pressure gradient correlates with SI severity and low albumin level in patients with portal hypertension [26, 27]. Further studies with larger sample sizes are needed to elucidate the mechanisms by which low CALLY scores are associated with poor prognosis in patients with GVs.

In this study, the cut-off value for the CALLY index was set at 4.35 using ROC analysis, and the HR for prognostic prediction was 3.61. Iida et al. set the cut-off value for the CALLY index as 5 as a prognostic indicator for HCC [9]; however, the cut-off values of the CALLY index reported for other cancers vary from 1.7 to 3.5, and no clear standard has been established. Furthermore, the AUC of 0.671 for the CALLY index in this study was comparable to the AUCs of 0.63-0.699 in other diseases, demonstrating the usefulness of the CALLY index [10-12]. In cirrhosis, Oikonomou et al. reported that the CAR was useful for predicting prognosis, with an AUC of 0.615 and HR of 2.082, using death or liver transplantation as the outcome [7]. Although an AUC of 0.671 indicated a moderate discriminatory ability, the AUC value of the CALLY index was the highest among the nutritional and inflammation-based indices in this study. Furthermore, multivariate analysis demonstrated that the CALLY index was significantly associated with a 3.8-fold higher risk of poor OS.

The CALLY index has been demonstrated to be an effective and simple prognostic indicator that can be easily calculated using only blood examination data, and it is necessary to establish a method for its application in clinical practice. In treatment for cancer, the CALLY index is considered a potential aid in determining indications for surgery and postoperative chemotherapy [28]. Our previous study showed that Sp + Dev improves liver function and hypersplenism and is a useful treatment option for GVs; however, it is more invasive than BRTO [4]. Therefore, for patients with a low CALLY index, non-surgical treatment may be preferable, and the CALLY index can assist in treatment decisions. The CALLY index also supports perioperative management; when Sp + Dev is planned, nutritional status should be improved, and inflammation should be reduced during the preoperative period to avoid a low CALLY index. Therefore, the surgical treatment of GVs requires the involvement of a multidisciplinary team that includes surgeons, hepatologists, and dieticians.

This study was limited by its small sample size, retrospective nature, and single-institution involvement, which may have resulted in biases related to patient backgrounds. To address these selection biases, survival analysis was performed after PSM, and the CALLY index was shown to have significant prognostic predictive ability. Since a standard cut-off value for the CALLY index has not been established, it may be necessary to discuss the differences in patient backgrounds when applying the results of this study to other patient populations. The lack of external validation is another limitation, and the generalizability of our findings remains to be confirmed. To overcome these limitations, future large-scale studies from multiple institutions are required, and our results may provide the basic clinical data.

## Conclusions

The CALLY index is an independent risk factor for poor OS in patients who underwent Sp + Dev for GVs caused by portal hypertension. Even after PSM to balance the background characteristics, a low CALLY index was significantly associated with poor prognosis, indicating that the CALLY index is a useful inflammation and nutritional biomarker for the prediction of prognosis for GVs after Sp + Dev. A low CALLY index was significantly correlated with high portal vein pressure after splenectomy, suggesting a potential association with the severity of portal hypertension. The results of this study may help in decision-making regarding the treatment of GVs and serve as valuable indicators for perioperative management.
